# Supplementary material for: Stomatal Opening Involves Polar, Not Radial, Stiffening Of Guard Cells
Source: Curr Biol. 2017 Oct 9;27(19):2974–2983.e2. doi: 10.1016/j.cub.2017.08.006 (PMC5640513; doi:10.1016/j.cub.2017.08.006)
Supplement: Document S1. Figures S1–S4 [file mmc1.pdf]

Current Biology, Volume *27*

Supplemental Information

Stomatal Opening Involves Polar, Not Radial,

Stiffening Of Guard Cells

Ross Carter, Hugh Woolfenden, Alice Baillie, Sam Amsbury, Sarah Carroll, Eleanor Healicon, Spyros Sovatzoglou, Sioban Braybrook, Julie E. Gray, Jamie Hobbs, Richard J. Morris, and Andrew J. Fleming

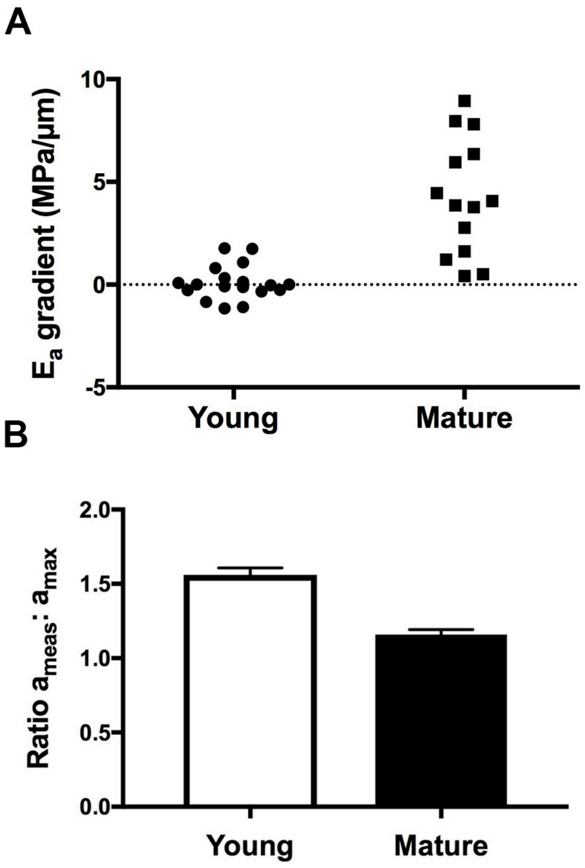

Figure S1. Related to Figure 1. Mature guard cells display a radial gradient in stiffness which is absent in young guard cells.

(A) Radial gradients (E_a/µm) calculated for individual guard cells. Stomata are classified as young (length:width ratio <1), giving guard cell n= 18, or mature (length:width ratio >1) giving guard cell n= 14. A Mann-Whitney test indicates that the mature guard cells displayed a significantly higher stiffness gradient than the young stomata ($p<0.001$). (B) Ratio of measured pore aperture (a_{meas}) to theoretical maximal aperture (a_{max}) for young or mature stomata after incubation under low CO_2 to open stomata. Error bars = sem.

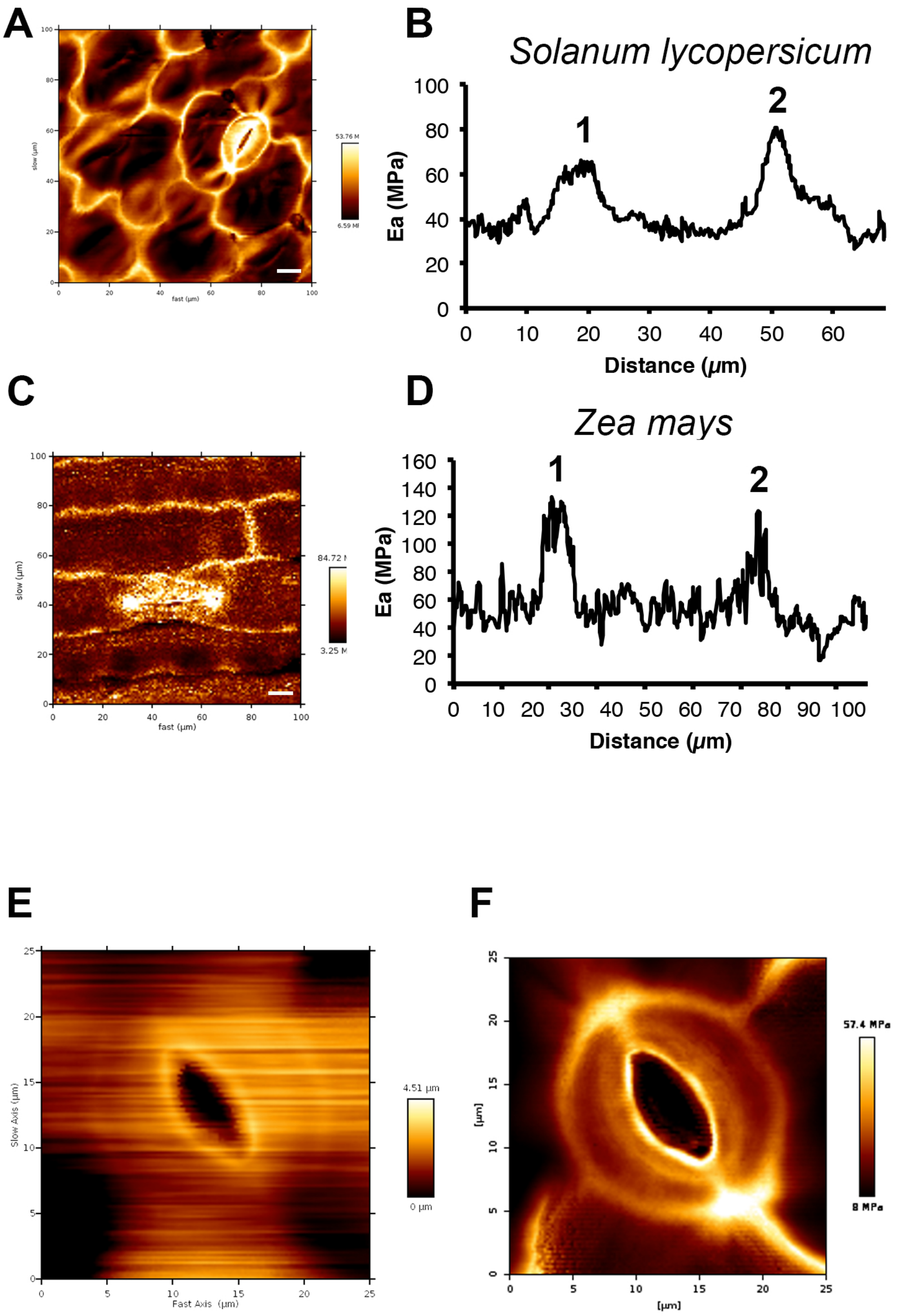

Figure S2. Related to Figure 1. Polar stiffening is observed in stomata from eudicot and monocot plants and does not reflect surface topography

(A) Force map of tomato (*Solanum lycopersicum*) epidermis showing the spatial pattern of Ea. (B) Distribution of Ea around the circumference of the stomatal complex shown in (A). Two main peaks of Ea are observed at the poles of the stomatal complex. (C) Force map of maize (*Zea mays*) epidermis showing the spatial pattern of Ea. (D) Distribution of Ea around the circumference of the stomatal complex shown in (C). Two main peaks of Ea are observed at the poles of the stomatal complex. Force maps and analyses presented are representative. Each analysis was performed at least three times with similar results. (E) Higher resolution topography and (F) Higher resolution force map of the Arabidopsis stomata shown in Figure 1I. In the vicinity of the guard cells there are no obvious topographical features corresponding to the polar localized regions of high Ea detected in (F). Scale bars A,C = 10µm.

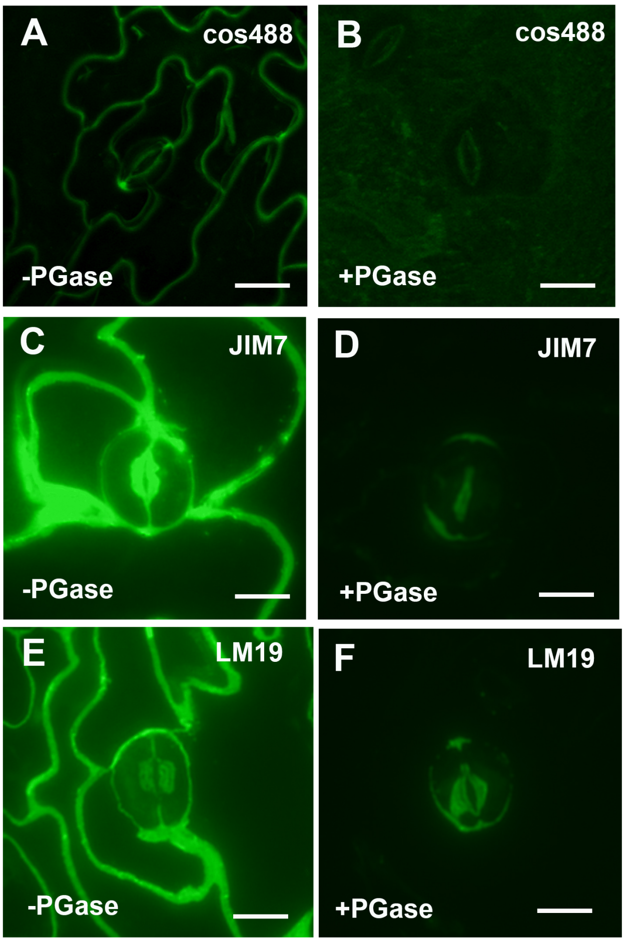

Figure S3. Related to Figure 4. Immunolabelling suggests that the stomatal poles have a specific pectin composition.

(A) Labeling with the COS^{488} probe reveals signal (green) in epidermal cell walls and peaks of signal intensity at the stomatal poles (as shown in Figure 4A) (B) Treatment of tissue with polygalacturonase (4h) leads to loss of COS^{488} binding throughout the epidermis. (C) Labelling with JIM7 antibody reveals signal (green) in all guard cell and epidermal walls, indicating the presence of pectin. (D) After treatment with polygalacturonase (4h) signal has disappeared from most walls but a weak signal is visible along the region of the cuticular ridge and in arcs around the stomatal poles. (E) Labelling with LM19 antibody, which detects de-esterified pectin, reveals a similar pattern to that observed in (C). (F) After treatment with polygalacturonase (4h) a pattern of LM19 binding similar to that observed in (D) is observed, with remnants of signal visible both along the inner radial walls and towards the stomatal poles. Images shown A-F are representative. Labellings were performed at least three times on independent biological samples with similar results being observed. Scale bars: A,B = 20 µm; (C-F) = 10 µm.

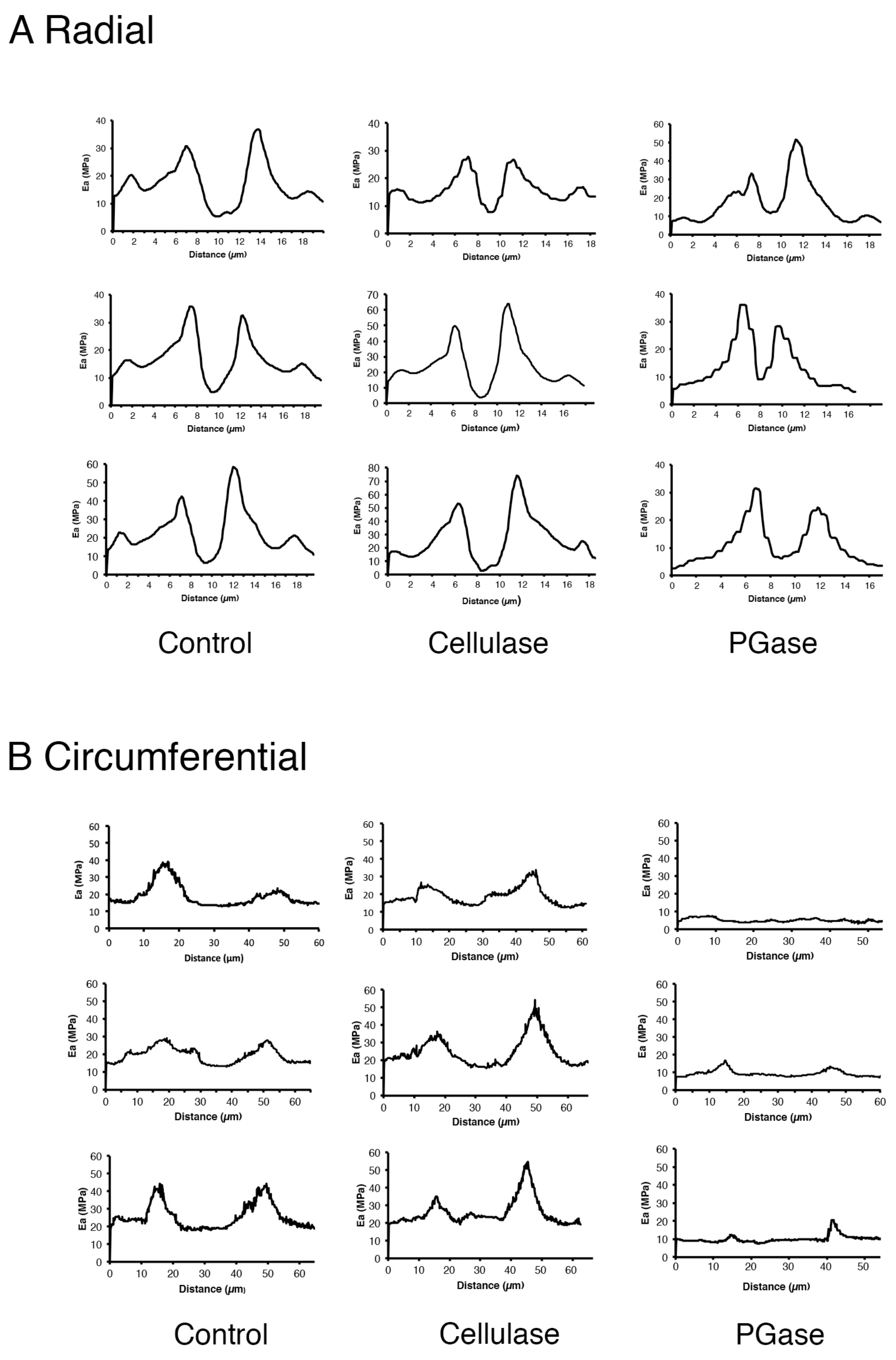

Figure S4. Related to Figure 4. Radial and circumferential Ea patterns in stomata treated with cell wall modifying enzymes

(A) Distribution of Ea across the diameter of the stomatal complexes (as indicated in schematic in Figure 1) after incubation for 4h in buffer (control), cellulase or polygalacturonase (PGase). Results are shown for 3 independent analyses for each treatment. The major peaks in Ea corresponding to the inner radial walls are clear in all samples but after PGase treatment the outer radial wall peaks are difficult to discern. (B) Distribution of Ea around the circumference (as indicated in schematic in Figure 1) of the stomatal complexes described in (A). Two main peaks of Ea corresponding to the poles of the stomatal complexes are apparent but after PGase treatment the peaks are more difficult to discern.
