# Supplementary material for: Stomatal Opening Involves Polar, Not Radial, Stiffening Of Guard Cells
Source: Curr Biol. 2017 Oct 9;27(19):2974–2983.e2. doi: 10.1016/j.cub.2017.08.006 (PMC5640513; doi:10.1016/j.cub.2017.08.006)
Supplement: Document S2. Article plus Supplemental Information [file mmc2.pdf]

Report

Current Biology

Stomatal Opening Involves Polar, Not Radial, Stiffening Of Guard Cells

Graphical Abstract

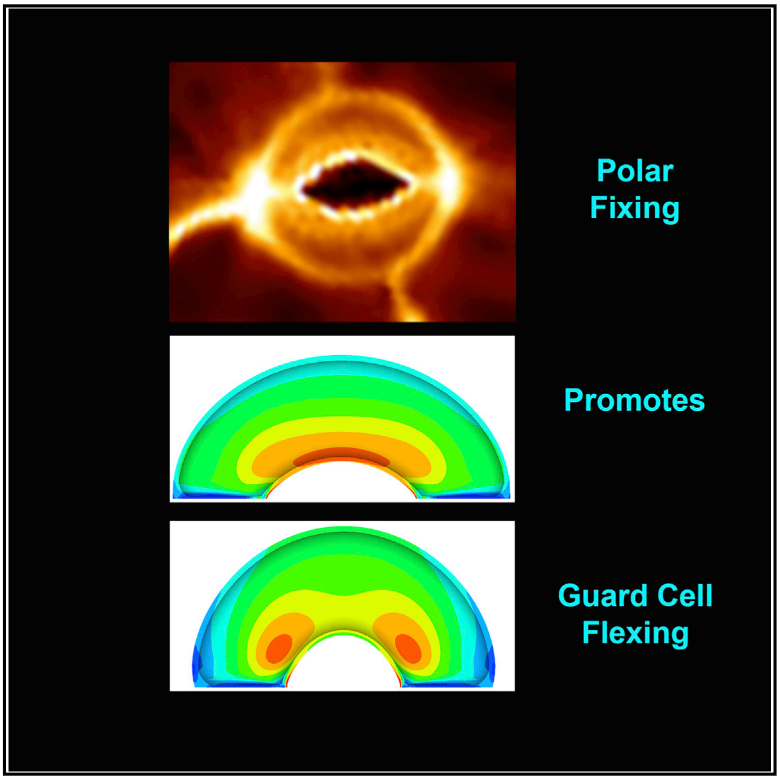

Highlights

- Stomatal poles are stiff and have a distinct cell wall composition
- Loss of polar stiffening is associated with decreased degree of stomatal opening
- Lack of radial guard cell stiffening does not preclude stomatal opening
- A “fix and flex” model predicts more efficient opening of stomata via polar stiffening

Authors

Ross Carter, Hugh Woolfenden, Alice Baillie, ..., Jamie Hobbs, Richard J. Morris, Andrew J. Fleming

Correspondence

a.fleming@sheffield.ac.uk

In Brief

Textbooks state that radial stiffening of guard cells is required for stomatal opening. Carter et al. show that this is not the case. They demonstrate that stomatal poles are stiffer than surrounding cells. A new “fix and flex” model is proposed in which polar stiffening leads to more efficient stomata, potentially allowing better water use by plants.

Carter et al., 2017, Current Biology *27*, 2974–2983
October 9, 2017 © 2017 The Authors. Published by Elsevier Ltd.
http://dx.doi.org/10.1016/j.cub.2017.08.006

Current Biology
Report

Stomatal Opening Involves Polar, Not Radial, Stiffening Of Guard Cells

Ross Carter,[1,2,3,6] Hugh Woolfenden,[3,6] Alice Baillie,[1] Sam Amsbury,[1] Sarah Carroll,[1] Eleanor Healicon,[1] Spyros Sovatzoglou,[2] Sioban Braybrook,[4] Julie E. Gray,[5] Jamie Hobbs,[2] Richard J. Morris,[3] and Andrew J. Fleming[1,7,*]
[1]Department of Animal and Plant Sciences, University of Sheffield, Sheffield, UK
[2]Department of Physics and Astronomy, University of Sheffield, Sheffield, UK
[3]Computational and Systems Biology, John Innes Centre, Norwich, UK
[4]Sainsbury Laboratory, Cambridge University, Cambridge, UK
[5]Department of Molecular Biology and Biotechnology, University of Sheffield, Sheffield, UK
[6]These authors contributed equally
[7]Lead Contact
*Correspondence: a.fleming@sheffield.ac.uk
http://dx.doi.org/10.1016/j.cub.2017.08.006

SUMMARY

It has long been accepted that differential radial thickening of guard cells plays an important role in the turgor-driven shape changes required for stomatal pore opening to occur [1–4]. This textbook description derives from an original interpretation of structure rather than measurement of mechanical properties. Here we show, using atomic force microscopy, that although mature guard cells display a radial gradient of stiffness, this is not present in immature guard cells, yet young stomata show a normal opening response. Finite element modeling supports the experimental observation that radial stiffening plays a very limited role in stomatal opening. In addition, our analysis reveals an unexpected stiffening of the polar regions of the stomata complexes, both in *Arabidopsis* and other plants, suggesting a widespread occurrence. Combined experimental data (analysis of guard cell wall epitopes and treatment of tissue with cell wall digesting enzymes, coupled with bioassay of guard cell function) plus modeling lead us to propose that polar stiffening reflects a mechanical, pectin-based pinning down of the guard cell ends, which restricts increase of stomatal complex length during opening. This is predicted to lead to an improved response sensitivity of stomatal aperture movement with respect to change of turgor pressure. Our results provide new insight into the mechanics of stomatal function, both negating an established view of the importance of radial thickening and providing evidence for a significant role for polar stiffening. Improved stomatal performance via altered cell-wall-mediated mechanics is likely to be of evolutionary and agronomic significance.

RESULTS AND DISCUSSION

Analysis of Stomatal Mechanical Properties Reveals Patterns of Cell Wall Modulus

Atomic force microscopy (AFM) was performed on leaves of *Arabidopsis* using a 5-nm-diameter pyramidal indenter on a cantilever of nominal 45 N/m stiffness mounted on a JPK Nano Wizard 3 instrument. Probing the surface generated force maps in which it was possible to identify stomata at various stages of development (Figure 1A) [5], ranging from guard mother cells (GMCs) undergoing the final symmetrical division to form two guard cells (Figure 1C), young stomata (characterized by an approximately equal length:width ratio) (Figure 1F), and more mature stomata (complex length greater than width; Figure 1I). Visual observation of the stiffness patterns indicated by apparent modulus values (E_a) suggested that although the more mature guard cells had the expected gradient of stiffness in which the inner radial region of each guard cell was stiffer than the outer radial part of the cell (Figure 1I), this pattern was not obvious in the younger stomata (Figure 1F). Quantitative analysis of E_a across the maximum diameter of stomata supported these observations. Thus, the E_a of mature stomata showed clear peaks in the inner radial regions of the guard cells relative to the outer radial regions (Figure 1J). A similar analysis of younger stomata did not reveal any such gradient (Figure 1G). By determining the difference in max E_a at the inner and outer radial regions across the width of the guard cells, values for E_a gradient were calculated (Figure S1A). For the more mature guard cells, the median E_a gradient was 4 MPa/μm (n = 14), whereas for younger guard cells, the median gradient was essentially 0 MPa/μm (n = 18). Statistical analysis using a Mann-Whitney test indicated that the mature guard cells displayed a significantly higher stiffness gradient ($p < 0.001$). We were able to analyze only two GMCs, and these showed a single peak of E_a in the center of the forming stomatal complex in the position of the dividing wall (Figure 1D). The value of the E_a for the dividing wall of GMCs was not higher than the outer cell wall of the GMCs, suggesting that there is no radial gradient of stiffness in the guard cells at formation.

To investigate whether the observed differences in radial E_a between young and mature guard cells reflected any difference in function, we performed bioassays on epidermal strips,

 © 2017 The Authors. Published by Elsevier Ltd.
This is an open access article under the CC BY license (http://creativecommons.org/licenses/by/4.0/).

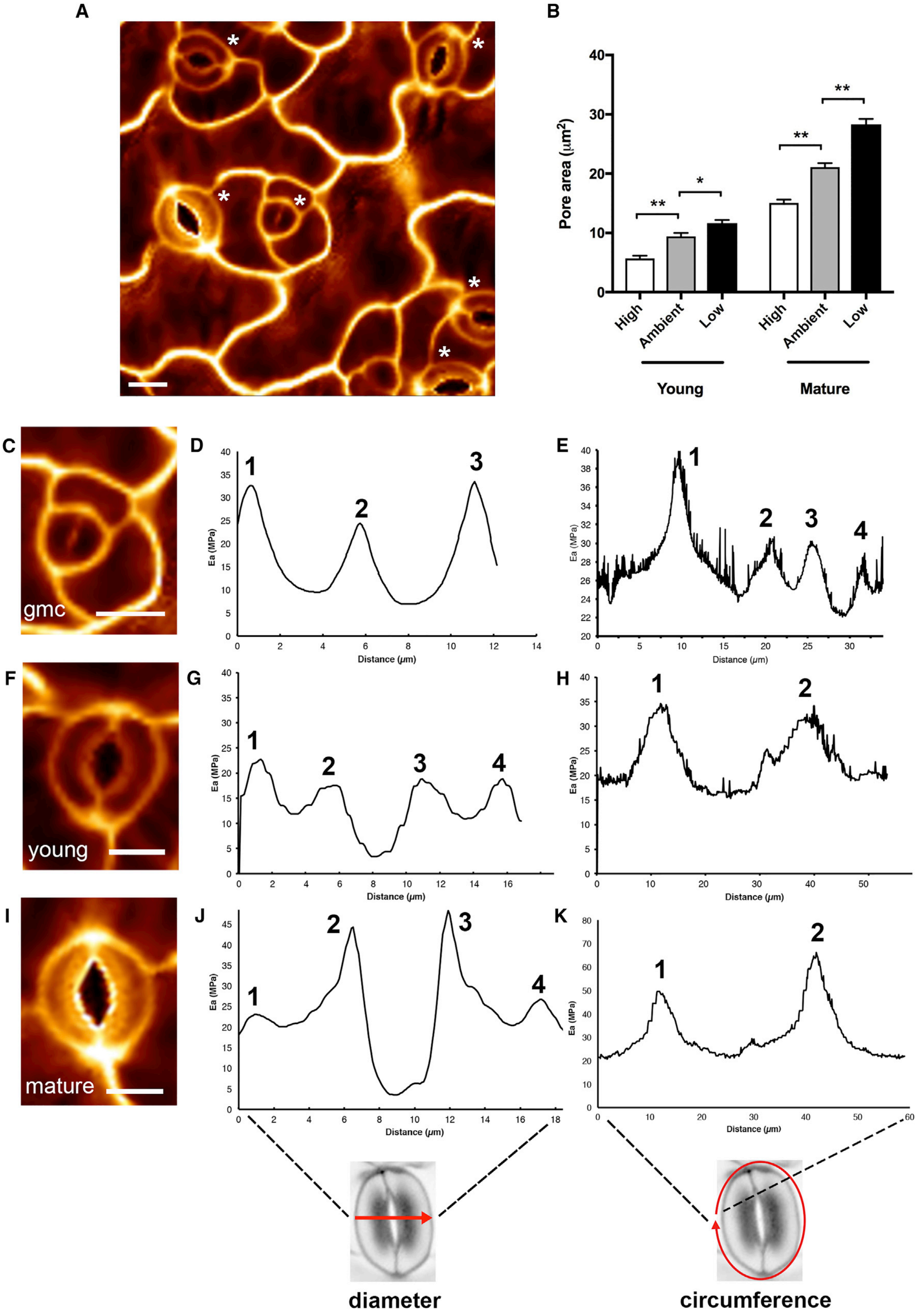

(legend on next page)

using depleted CO_2 to trigger stomatal opening and elevated CO_2 to close stomata [6]. These results indicated that both young and mature stomata are able to open and close in response to an external trigger (Figure 1B). The absolute values for pore aperture were clearly lower for young stomata compared with mature stomata. Comparison of the measured maximal pore aperture attained under low CO_2 conditions with the theoretical maximal aperture predicted from pore geometry indicated that the younger stomata were just as capable as mature stomata of opening their pores; thus, the lower absolute values for pore aperture most likely simply reflected stomatal size differences between young and mature stomata (Figure S1B).

To extend our understanding of the physics of stomatal opening/closing, we exploited a recently developed finite element model (STAR Methods). In the baseline model, the guard cells have a circular cross-section and uniform wall thickness and, thus, uniform mechanical properties (Figure 2A). Under these conditions, as the epidermal and internal pressure (turgor) of the guard cells are increased from zero, the system moves slightly away from the starting geometry, but even as the guard cell turgor pressure rises above the epidermal pressure (limited here to 0.5 MPa), there is initially no increase in pore aperture (Figure 2B). When the guard cell turgor pressure reaches about 1.3 MPa, the stomatal aperture starts to increase, approaching a maximum as pressure increases above 5 MPa. When the model is adjusted so that the cells have a geometry more in keeping with that described in the literature [7], leading to differential wall thickness along the inner radial wall (variable wall thickness, VWT model) (Figure 2A), there is a slight shift in the aperture-response curve, favoring larger aperture at a lower pressure and smaller aperture at higher pressure, but the changes are relatively small (Figure 2B). When we explored the sensitivity of the VWT model to altered wall thickness, there was a very limited response to this parameter. Thus, increasing or decreasing inner wall thickness in the VWT model by 10% had essentially no outcome on the aperture/pressure response curve (Figure 2C).

An increased thickening of the inner radial wall of guard cells was observed in early botanical studies, leading to the widely accepted view that this leads to a stiffening of the wall, which is required for the curling displayed by guard cells as they expand due to increased turgor pressure [1–4]. Although this interpretation has been challenged [8, 9], lack of measurement of guard cell mechanics has limited the scope for discussion. AFM provides a means of assessing mechanical properties that has become increasingly used in the analysis of biological material, including plants [10–13]. Although care must be taken in the interpretation of such data (since the values obtained are influenced by a range of factors, including the geometry and mechanical properties of the tips used, and factors intrinsic to the complex composition and geometry of the tissue), AFM provides a robust method for estimating relative stiffness across cellular dimension [13–16]. We report stiffness as an apparent modulus, E_a, not inferring a specific modulus of the material being indented. Our results support the interpretation that the observed thickening of the inner radial wall leads to a gradient of stiffening across the guard cell [1–4]; however, this gradient is only observed in relatively mature cells. Younger guard cells do not display any consistent gradient of radial stiffening, yet our measurements of pore aperture indicate that these stomata are able to respond to appropriate triggers by opening the stomatal pore at least as widely as the calculated theoretical maximum (Figure S1B). Coupled with our modeling indicating that increased stiffening of the inner radial wall has a minimal outcome on stomatal movement, we propose that radial stiffening of guard cells is not required for stomatal opening.

This raises the question of what function it might play. The finite element modeling approach allows prediction of strain/stress patterns within the guard cells as they undergo movement. These data indicate that, in the baseline model, large gradients of strain/stress are generated across the inner radial wall of the guard cell during stomatal opening (Figures 2E

Figure 1. Stomata Show Stage-Dependent Patterns of Modulus

(A) Force map of a leaf epidermis showing the spatial pattern of E_a. Stomata (indicated by asterisks) at different stages of differentiation are distributed across the epidermis and show different patterns of E_a, indicated by relative signal value (yellow, high; red/black, low).

(B) Bioassays of young and mature stomata indicate that they both respond to low CO_2 by increasing pore area and to high CO_2 by decreasing pore area. Single asterisk indicates significant difference $p < 0.01$, $n > 23$; double asterisk indicates significant difference $p < 0.001$, $n > 23$ (ANOVA was performed on "young" or "mature" datasets, followed by a Tukey test). Error bars indicate SEM.

(C) Force map of a guard mother cell (GMC) showing the symmetrical cross wall separating the two daughter guard cells.

(D) Distribution of E_a across the diameter (as shown in schematic) of the GMC shown in (C). Three peaks of E_a of similar value are detected, corresponding to the three walls of the GMC.

(E) Distribution of E_a around the circumference of the GMC shown in (C), with the start point at the equator (as shown in the schematic). A series of peaks of E_a are observed.

(F) Force map of a young stomata consisting of two separated guard cells.

(G) Distribution of E_a across the diameter of the stomatal complex shown in (F). Four peaks are detected, corresponding to the pairs of walls defining the guard cells. The maximum peak value is similar for all four walls.

(H) Distribution of E_a around the circumference of the stomatal complex shown in (F). Two main peaks of E_a are observed at the poles of the stomatal complex. The shoulder on the second peak corresponds to the junction with the epidermal cell on the right-hand guard cell.

(I) Force map of a mature stomata consisting of two guard cells.

(J) Distribution of E_a across the diameter of the stomatal complex shown in (I). Four peaks are detected, corresponding to the pairs of walls defining the guard cells. The maximum E_a value for the inner radial walls is higher than the peak E_a for the outer radial walls.

(K) Distribution of E_a around the circumference of the stomatal complex shown in (I). Two main peaks of E_a are observed at the poles of the stomatal complex. The minor third peak corresponds to the junction with the epidermal cell on the right-hand guard cell. Representative images and analyses of young (F–H) and mature (I–K) guard cells are shown. Force maps were obtained from a total of 14 young and 18 mature guard cells. Scale bars in (A), (C), (F), and (I), 10 μm.

See also Figures S1 and S2.

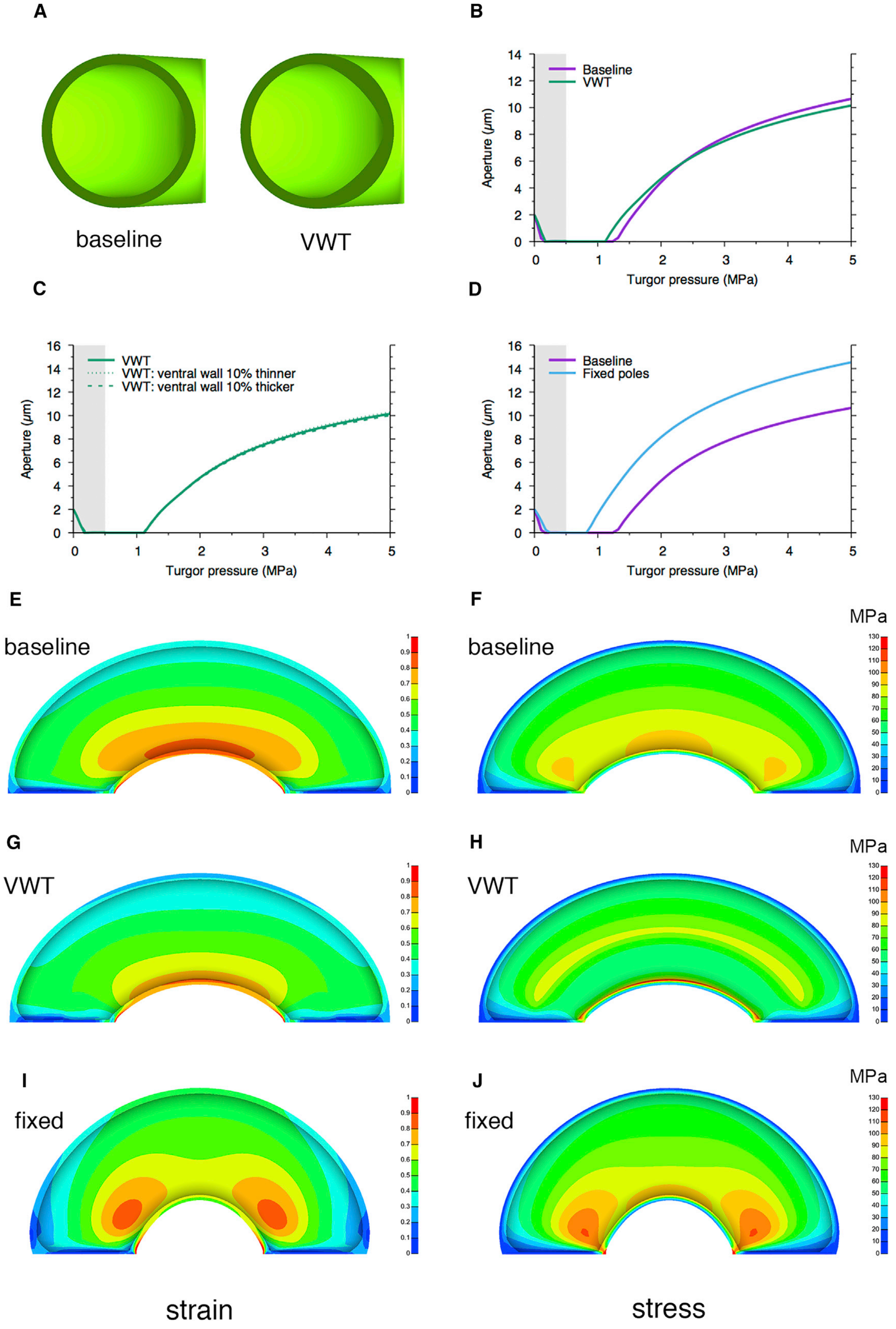

(legend on next page)

and 2F). The geometry (and associated differential wall thickening) in the VWT model leads to a non-intuitive dissipation of these strain/stress gradients so that the maximum stress occurs away from the inner radial wall and the stress gradient is diminished (Figures 2G and 2H). Due to the vital role that stomata play in the control of plant gas and water relations, they must repeatedly adjust their aperture to the ambient environment [17]; thus, the guard cells must undergo extensive and repeated strain. We suggest that the differential thickening of the inner radial wall in mature stomata generated as a consequence of guard cell geometry acts primarily to alleviate the potential for mechanical failure, helping to maintain cell wall integrity as it undergoes repeated stress/strain cycles. This structural modification has an associated outcome of slightly altering the aperture response to turgor pressure.

Polar Stiffening Modulates Stomatal Function

An unexpected observation from our analysis was the apparent stiffening of the polar regions of both young (Figure 1H) and mature (Figure 1K) stomata. As far as we are aware, this has not previously been observed. An analysis of tomato and maize leaves revealed comparable patterns of stiffening, suggesting that this phenomenon might be widespread (Figures S2A–S2D), and higher-resolution imaging did not reveal any overt surface features that might lead to such localized regions of high E_a (Figures S2E and S2F). To investigate the function of such polar stiffening, we further explored the model described in Figure 2. As shown in Figure 3A, both the baseline and VWT models predict that as turgor pressure increases (and, as a consequence, pore width increases), stomatal complex length should increase. However, analysis of samples incubated under differing CO_2 concentrations to open or close the stomatal pore indicated no trend for change in complex length at different pore widths (Figure 3B), as also observed by other authors [18]. This is in contrast to measured pore length, which showed a strong positive correlation with pore width under the same treatments ($p < 0.0001$, $n = 360$) (Figure 3C). The experimental data suggested to us that the measured local stiffening observed in Figures 1F and 1I might reflect a pinning down of the poles so that complex length does not change during opening/closure of the stomata. We therefore modified the model to impose a restriction on stomatal complex length change during pore opening/closure (blue line in Figure 3A), better capturing experimental reality. This had a dramatic outcome on aperture change in response to increase in turgor pressure, with opening occurring at a lower pressure, a greater increase in aperture per unit pressure being achieved, and a larger final aperture being attained ("fixed poles" curve in Figure 2D).

To investigate the molecular structure of the stomatal poles that might underpin the observed stiffening, we took an *in situ* labeling approach to characterize the spatial pattern of cell wall epitopes. Recent work has identified a chitosan oligosaccharide (COS^{488}) probe that enables localization of de-esterified homogalacturonic polymers in plant cell walls [19]. Incubation of this probe with intact leaf epidermal tissue revealed binding to the epidermal pavement cells and especially strong signal at the stomatal poles, with apparent exclusion from the outer radial walls of the guard cells (Figure 4A; Figure S3A). Treatment of tissue with polygalacturonase led to loss of COS^{488} binding (Figure 4B; Figure S3B), corroborating that the probe was detecting a pectin motif in this region. Our previous work using antibodies raised against pectins revealed that guard cell walls are distinguished by the exclusion of epitopes corresponding to methylated pectin and the accumulation of epitopes corresponding to de-esterified pectin [20]. Interestingly, following treatment with polygalacturonase, the uniform signal observed around guard cells with antibodies JIM7 and LM19 (which detect general levels of pectin and de-esterified pectin, respectively [21]) was replaced by a pattern of weak signal around the stomatal poles (Figures S3C–S3F).

Figure 2. Finite Element Modeling Indicates Only a Minor Role for Radial Stiffening in Stomatal Function but Demonstrates that Fixing Stomatal Poles Has a Major Influence on Aperture Response to Change of Turgor Pressure

(A) Cross-sections through guard cells modeled using the baseline parameters (circular cross-section and uniform wall thickness) or the variable wall thickness (VWT) model in which a rounded triangular geometry leads to differential inner wall thickness. The cell wall is modeled as an anisotropic material, parameterized by cellulose micro-fibrils embedded in an isotropic matrix. The micro-fibrils are oriented circumferentially in all models.

(B) Modeled relationship of stomatal aperture to guard cell turgor pressure. In the baseline model (purple), aperture increases as pressure increases above about 1.3 MPa, reaching a maximum value as pressure exceeds 5 MPa. Both epidermal and guard cell turgor are increased initially (gray area) after which only guard cell turgor increases. Modification of the model to include a VWT (shown in A) leads to a slight alteration in curve shape (green) so that opening occurs at a slightly lower turgor pressure and the maximal aperture attained is slightly lower.

(C) Exploration of the VWT model by increasing or decreasing the inner (ventral) wall thickness by 10% indicates essentially no outcome on the aperture/pressure response curve (lines superimposed).

(D) Modification of the baseline model (purple) so that the poles of the guard cells are fixed to prevent stomatal complex elongation leads to a modified output curve (blue) in which pore opening occurs at a lower turgor pressure and the final aperture attained is larger than the baseline model.

(E) Effective Lagrange strain for the inside of a guard cell modeled using the baseline parameters. The colored scale indicates the range of strain calculated in different regions of the cell, with a gradient of strain occurring across the cell radius with the inner radial wall having a high strain.

(F) Effective stress pattern in the guard cell modeled in (E). A radial stress pattern is generated with high stress at points along the inner radial wall.

(G) As in (E) but with VWT parameters used in the model. A decreased strain gradient occurs across the cell.

(H) Effective stress pattern in the guard cell modeled in (G). The stress pattern observed in (F) is dissipated so that less extreme gradients are formed, with maximal stress occurring in a medial region.

(I) As in (E) but with the stomatal poles fixed (as in D). The pattern is modified from (E) so that high strain gradients form in localized regions toward the guard cell poles.

(J) Effective stress pattern in the guard cell modeled in (I). Steeper stress gradients now form toward the guard cell poles compared to (F). In (E), (G), and (I) the strain is dimensionless and is capped at 1 for consistency across the figures. Only the regions immediately neighboring the point at which the pore adjoins the polar wall exceed this value. Strain is a dimensionless tensor describing the deformation of the material, which in simple cases is defined as length change per length. Stress is a tensor which characterizes the internal forces within a material as force per area. In (F), (H), and (J) the unit of stress is MPa, where $1\ Pa = 1\ Nm^{-2}$.

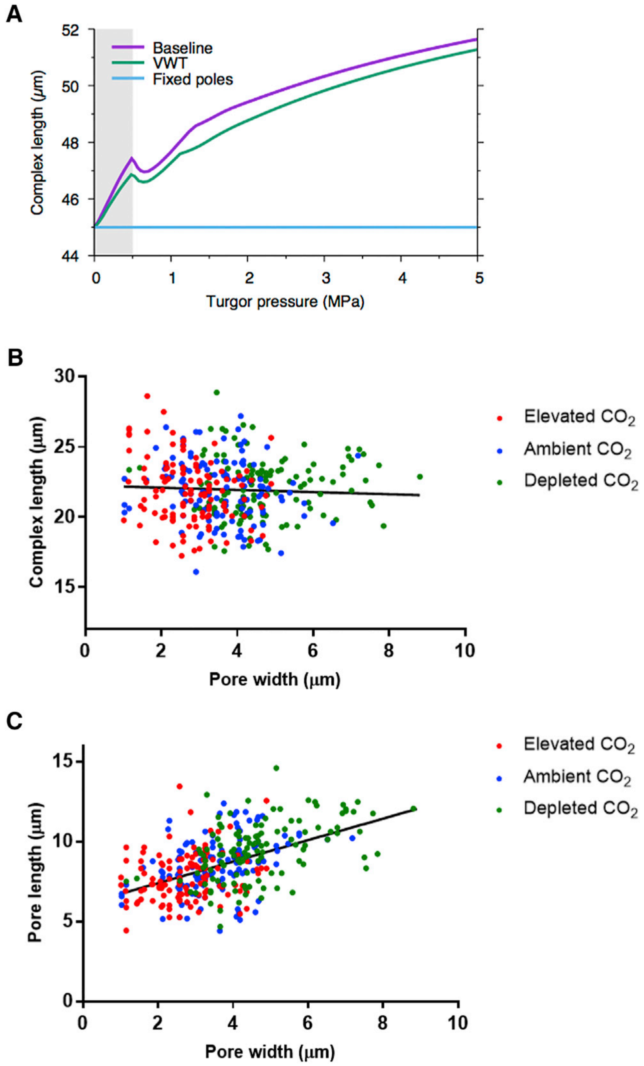

Figure 3. Measured Change in Stomatal Dimensions during Opening and Closing Supports a Fixed Position of the Stomatal Poles

(A) Modeled change in stomatal complex length with increase in guard cell pressure predicts a gradual increase in length at pressures above 1 MPa, both for the baseline (purple) and the VWT model (green), whereas the fixed pole model imposes a constant complex length (blue).

(B) Measured complex length in mature stomata triggered to close by elevated CO_2 (red), open by depleted CO_2 (green), or incubated under ambient CO_2 levels (blue). Complex length does not overtly change relative to pore width. Regression analysis was used to calculate the line indicated but is supported with only a low confidence value ($p = 0.354$, $n = 360$), suggesting a very limited relationship of complex length and pore width.

(C) Measured pore length in mature stomata triggered to close by elevated CO_2 (red), open by depleted CO_2 (green), or incubated under ambient CO_2 levels (blue). Pore length increases with pore width. Regression analysis was used to calculate the line indicated, which is supported with $p < 0.0001$ ($n = 360$), suggesting a close relationship of pore length and pore width.

Note that the size parameters used for the model are based on those from the literature for *Vicia faba*, thus the absolute magnitudes of stomatal complex length are greater in (A) than in (B).

Although both COS[488] and LM19 detect de-esterified pectin, it is likely that the signal observed depends on the degree of de-esterification and the local matrix conformation, which may restrict probe access [19, 22], complicating interpretation of the patterns in signal observed. As a consequence of such technical challenges, our detailed understanding of plant cell wall molecular architecture is still somewhat limited [23, 24]. However, taken together, the data in Figure 4A and Figure S3 are consistent with the hypothesis that stomatal poles in *Arabidopsis* have a distinct cell wall pectin structure, which might define the localized regions of stiffness detected in our AFM analysis. Modeling of the fixed pole model indicated that it would lead to an altered pattern of strain/stress during stomatal opening, with a focusing of gradients toward the polar regions of the guard cells (Figures 2I and 2J). Whether guard cell wall composition/structure is modified in these regions to cope with these predicted strain/stress patterns awaits further analysis.

To investigate whether the localized difference in pectin structure was related to the observed polar stiffening, and thus the role of polar stiffening in stomatal function, we treated leaf explants with cell-wall-modifying enzymes [25]. Treatment of tissue with buffer alone did not overtly change the pattern of stiffness observed in mature stomata (Figure 4C). Quantitative analysis of E_a across the diameter and around the circumference of mature stomata revealed normal patterns, with a radial gradient in the guard cells and two peaks of E_a in the polar regions (Figures 4D and 4E). Similarly, treatment with exogenous cellulase for 4 hr did not alter the stiffness patterns in a major fashion from those observed in control tissue (Figures 4F–4H). However, treatment with polygalacturonase led to major changes in stiffness pattern. With respect to the stomata, there was an accentuation in the apparent relative gradient of radial stiffening of the guard cells, and polar stiffening was less marked (Figure 4I). Quantitation of the radial and circumferential patterns of E_a substantiated these observations. Thus, the E_a peaks corresponding to the outer radial walls of the guard cells tended to be diminished (Figure 4J; Figure S4A), and the polar peaks of E_a tended to be narrower and much smaller in absolute value (Figure 4K; Figure S4B).

We performed opening/closing assays to test the outcome of enzyme treatment on stomatal function. After all treatments, guard cells retained the ability to increase pore aperture following exposure to depleted (low) CO_2 levels (Figure 4B); however, the basal aperture under ambient conditions was significantly lower in the polygalacturonase-treated stomata than in those treated with cellulase or buffer alone (ANOVA with post hoc Tukey, $p < 0.001$, $n = 40$, experiment repeated three times). The maximal aperture achieved by both polygalacturonase- and cellulase-treated stomata was smaller than that achieved in control tissue (ANOVA with post hoc Tukey, $p < 0.05$, $n = 40$).

A decrease in stomatal pore aperture relative to control after enzyme treatment could occur via a number of mechanisms. For example, treatment with polygalacturonase led to an altered gradient of stiffness across guard cells (Figures 4I and 4J), but our finite element modeling suggested that alteration in radial stiffness has only a very moderate effect on stomatal opening (Figure 2C). It was also apparent that treatment with polygalacturonase led to a decreased relative stiffness in all epidermal cell walls (compare Figure 4I with Figures 4C and 4F); however, it is not obvious how such a change would lead to a decrease in pore aperture under ambient conditions. Epidermal cells

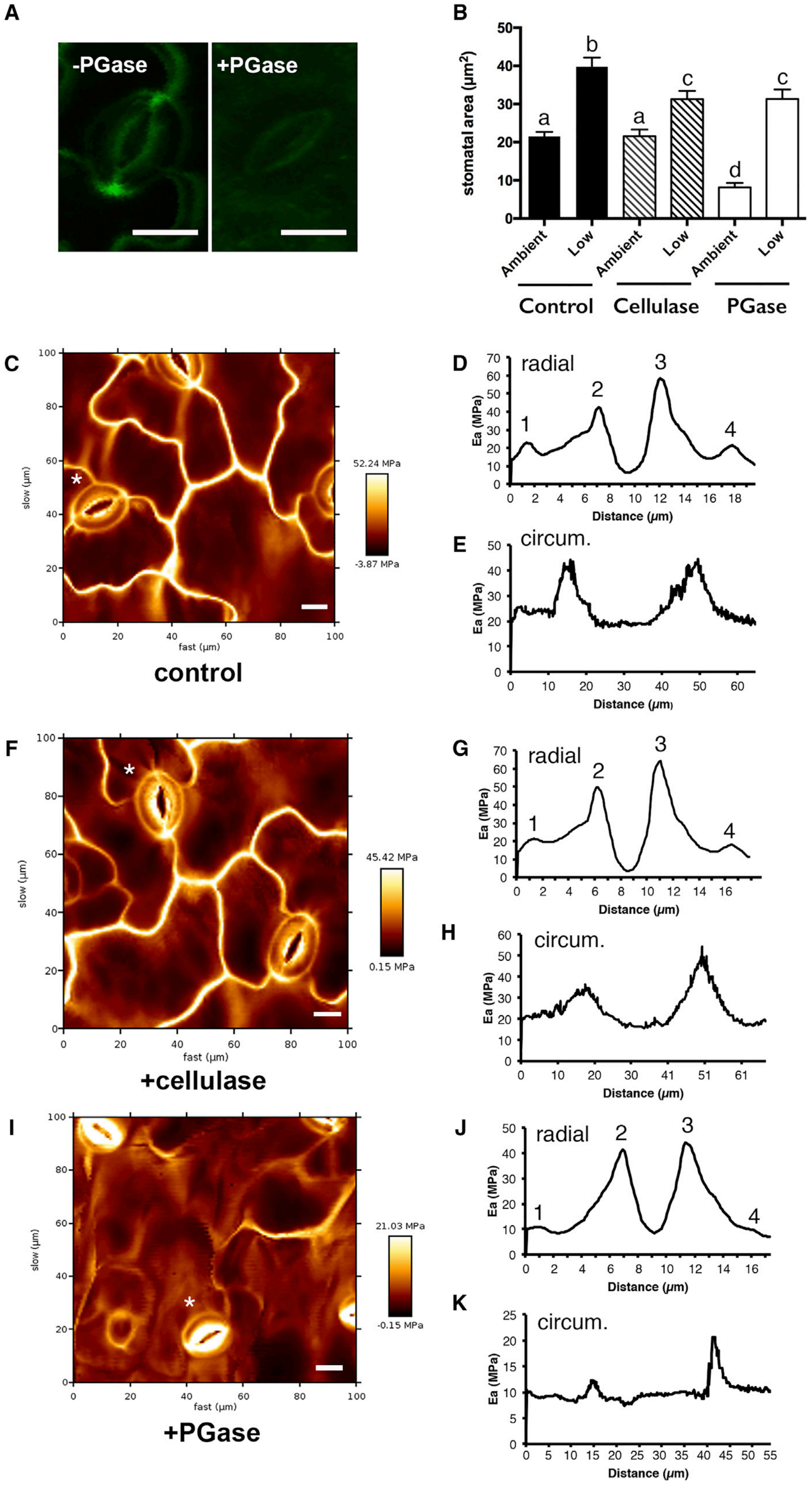

(legend on next page)

surrounding stomata are expected to exert a mechanical advantage [26, 27], so, if anything, weakening of these supporting cells might lead to an increased pore aperture for any given guard cell turgor pressure, the opposite of the phenotype observed. Decreased pressure within the guard cells under ambient conditions would obviously lead to a decreased pore aperture, but it is not apparent why this would be a primary outcome following treatment with polygalacturonase (and which was not observed after cellulase treatment). A final possibility is that the loss of polar stiffening observed after polygalacturonase treatment (Figure 4K; Figure S4B) underpins the shift in stomatal dynamics. Considering the pore aperture response to altered turgor pressure depicted for the baseline and fixed pole models (Figure 2D), it is clear that, above 1 MPa, a loss of polar stiffening leads to a large decrease in aperture for any given pressure as the stomatal dynamics shift from the "fixed poles" to the "baseline" curve. This would account for the decreased aperture under ambient conditions recorded in stomata treated with polygalacturonase (Figure 4B). It should be noted that our model predicts that after loss of polar stiffening and consequent shift to the baseline model, stomata are still able to open, but the final aperture is expected to be smaller than in the fixed poles model. The experimental data in Figure 4B support this prediction. Cellulase treatment of stomata led to results intermediate between control and polygalacturonase-treated samples (Figure 4B). There was no evidence of decreased aperture under ambient conditions, but the maximal aperture obtained under conditions favoring opening was lower than control. We suspect that this decrease in maximal aperture might reflect a gradual loss in tissue integrity after cellulase treatment, as previously observed [28]. Overall, our observations are consistent with the proposal that polar stiffening, mediated at least in part by localized accumulation of de-esterified pectin, plays a role in stomatal function. Stiffening of guard cell poles limits stomatal complex extension under opening conditions, leading to a mechanical system that shows a greater response in pore aperture per change in guard cell pressure.

Such a system would be expected to be evolutionarily advantageous. Plants adapt stomatal aperture to changing environments, and limits in the rapidity with which they can do this leads to inefficiencies [17]. Indeed, it has been proposed that one of the reasons for the evolutionary success of some plant groups is that their stomata have evolved to be able to respond more rapidly to changing environment [26]. Whether the structure of the guard cell wall in the stomatal poles has played an evolutionary role in improving stomatal efficiency awaits elucidation, but our work sets the foundation for this future research. Due to the importance of stomata in plant water relationships, a deeper understanding of the properties of guard cell walls in setting the mechanical response to external triggers may also help in the selection and engineering of improved crops [20, 29, 30].

In conclusion, the results reported here negate a widely held view on the importance of radial guard cell wall thickening in stomatal opening, provide an alternative view on the importance of guard cell geometry in dissipating cell wall stress gradients, and identify polar stiffening of stomata as a potentially widespread phenomenon that leads to improved stomatal response to altered guard cell turgor pressure.

STAR★METHODS

Detailed methods are provided in the online version of this paper and include the following:

- KEY RESOURCES TABLE
- CONTACT FOR REAGENT AND RESOURCE SHARING

Figure 4. Polar Cell Wall Structure Plays a Role in Stiffening and Stomatal Function

(A) Labeling of stomata with the COS^{488} probe reveals a high level of signal (green) at the stomatal poles (left). Treatment of tissue with polygalacturonase (4 hr) leads to loss of COS^{488} binding (right).

(B) Bioassays after pre-treatment with buffer (control), cellulose, or polygalacturonase (PGase) indicate that stomata retain the ability to open in response to low CO_2 after all treatments, but the stomatal aperture attained after PGase treatment is significantly smaller, both at ambient and low CO_2, relative to the control. ANOVA was performed across all samples with post hoc Tukey. Columns indicated with the same letter cannot be distinguished from each other at the 0.05 confidence limit (n = 40). Error bars indicate SEM.

(C) Force map of epidermis from a control sample showing the spatial pattern of E_a after 4 hr incubation of tissue in buffer. Relative signal value is indicated by high (yellow) to low (red/black).

(D) Distribution of E_a across the diameter (as shown in schematic in Figure 1) of the stomata indicated by asterisk in (C). Four peaks are detected, corresponding to the pairs of walls defining the guard cells. The maximum peak value for the inner radial walls (peaks 2 and 3) is higher than the peak value for the outer radial walls (peaks 1 and 4).

(E) Distribution of E_a around the circumference (as shown in schematic in Figure 1) of the stomatal complex shown in (C). Two main peaks of E_a are observed at the poles of the stomatal complex.

(F) Force map of epidermis showing the spatial pattern of E_a after 4 hr incubation of tissue in cellulase. Relative signal value is indicated by high (yellow) to low (red/black).

(G) Distribution of E_a across the diameter of the stomata indicated by asterisk in (F). Four peaks are detected, corresponding to the pairs of walls defining the guard cells. The maximum E_a for the inner radial walls is higher than the peak value for the outer radial walls.

(H) Distribution of E_a around the circumference of the stomatal complex shown in (F). Two main peaks of E_a are observed at the poles of the stomatal complex.

(I) Force map of epidermis showing the spatial pattern of E_a after 4 hr incubation of tissue in polygalacturonase. Relative signal value is indicated by high (yellow) to low (red/black).

(J) Distribution of E_a across the diameter of the stomata indicated by asterisk in (I). Two broad, asymmetric peaks are detected, with the highest values at the inner radial walls of the two guard cells. The peaks corresponding to the outer radial wall (peaks 1 and 4) are only barely detectable.

(K) Distribution of E_a around the circumference of the stomatal complex shown in (I). Two main peaks of E_a are observed at the poles of the stomatal complex. The E_a value of these peaks is lower than those observed in (E) and (H). Representative images and analysis are shown for control (C–E), cellulase (F–H), and polygalacturonase-treated tissue (I–K). The analyses were repeated at least three times with similar results (data shown in Figure S4). Scale bars in (A), (C), (F), and (I), 10 μm.

See also Figures S3 and S4.

- EXPERIMENTAL MODEL AND SUBJECT DETAILS
  - Plant Growth and Tissue Treatments
- METHOD DETAILS
  - Stomatal Aperture Measurements
  - Immunolabeling
  - Atomic Force Microscopy
  - Modeling
- QUANTIFICATION AND STATISTICAL ANALYSIS
- DATA AND SOFTWARE AVAILABILITY

SUPPLEMENTAL INFORMATION

Supplemental Information includes four figures and can be found with this article online at http://dx.doi.org/10.1016/j.cub.2017.08.006.

AUTHOR CONTRIBUTIONS

Conceptualization, A.J.F., J.H., and R.J.M.; Investigation, R.C., H.W., A.B., S.A., S.C., E.H., A.J.F, and S.S.; Writing–Original Draft, A.J.F.; Writing–Review & Editing, all authors; Supervision, A.J.F., J.E.G., J.H., R.J.M., and S.B.; Funding Acquisition, A.J.F., J.E.G., S.B., J.H., and R.J.M.

ACKNOWLEDGMENTS

The work reported here was funded by a Leverhulme Research Fellowship (A.J.F.); White Rose BBSRC-DTP awards (BB/M01151 and BB/J014443) to S.C., E.H., S.A., A.J.F., and J.E.G.; and the Gatsby Foundation (A.B. and S.B.). R.J.M. and H.W. acknowledge support from BBSRC's Institute Strategic Programme on Biotic Interactions underpinning Crop Productivity (BB/J004553/1) and Plant Health (BB/P012574/1). The Microscopy Facility at the Sainsbury Laboratory is supported by the Gatsby Charitable Foundation. We thank William Willats (University of Newcastle) for the kind gift of the COS^{488} probe.

Received: April 20, 2017
Revised: June 14, 2017
Accepted: August 2, 2017
Published: September 21, 2017

REFERENCES

1. Mohl, H.v. (1856). Welches ursachen bewirken die erweiterung und verengung fer spaltoeffnungen. Bot. Z. *14*, 713–721.
2. Esau, K. (1967). Plant Anatomy, Second Edition (John Wiley).
3. Smith, A.M., Coupland, G., Dolan, L., Harberd, N., Jones, J., Martin, C., Sablowski, R., and Amey, A. (2010). Plant Biology (Garland Science).
4. Strasburger, E., Noll, F., Schenck, H., and Schimper, A.F.W. (1898). A Text-book of Botany (Macmillan).
5. Pillitteri, L.J., and Torii, K.U. (2012). Mechanisms of stomatal development. Annu. Rev. Plant Biol. *63*, 591–614.
6. Kim, T.H., Böhmer, M., Hu, H., Nishimura, N., and Schroeder, J.I. (2010). Guard cell signal transduction network: advances in understanding abscisic acid, CO2, and Ca2+ signaling. Annu. Rev. Plant Biol. *61*, 561–591.
7. Spence, R.D., Wu, H., Sharpe, P.J.H., and Clark, K.G. (1986). Water-stress effects on guard-cell anatomy and the mechanical advantage of the epidermal cells. Plant Cell Environ. *9*, 197–202.
8. Aylor, D.E., Parlange, J.Y., and Krikorian, A.D. (1973). Stomatal mechanics. Am. J. Bot. *60*, 163–171.
9. Cooke, J.R., Debaerdemaeker, J.G., Rand, R.H., and Mang, H.A. (1976). Finite-element shell analysis of guard cell deformations. Trans. ASAE *19*, 1107–1121.
10. Peaucelle, A., Braybrook, S.A., Le Guillou, L., Bron, E., Kuhlemeier, C., and Höfte, H. (2011). Pectin-induced changes in cell wall mechanics underlie organ initiation in Arabidopsis. Curr. Biol. *21*, 1720–1726.
11. Sampathkumar, A., Krupinski, P., Wightman, R., Milani, P., Berquand, A., Boudaoud, A., Hamant, O., Jönsson, H., and Meyerowitz, E.M. (2014). Subcellular and supracellular mechanical stress prescribes cytoskeleton behavior in Arabidopsis cotyledon pavement cells. eLife *3*, e01967.
12. Milani, P., Gholamirad, M., Traas, J., Arnéodo, A., Boudaoud, A., Argoul, F., and Hamant, O. (2011). In vivo analysis of local wall stiffness at the shoot apical meristem in Arabidopsis using atomic force microscopy. Plant J. *67*, 1116–1123.
13. Braybrook, S.A., and Jönsson, H. (2016). Shifting foundations: the mechanical cell wall and development. Curr. Opin. Plant Biol. *29*, 115–120.
14. Cosgrove, D.J. (2016). Plant cell wall extensibility: connecting plant cell growth with cell wall structure, mechanics, and the action of wall-modifying enzymes. J. Exp. Bot. *67*, 463–476.
15. Peaucelle, A., Wightman, R., and Höfte, H. (2015). The control of growth symmetry breaking in the Arabidopsis hypocotyl. Curr. Biol. *25*, 1746–1752.
16. Braybrook, S.A., and Peaucelle, A. (2013). Mechano-chemical aspects of organ formation in Arabidopsis thaliana: the relationship between auxin and pectin. PLoS ONE *8*, e57813.
17. McAusland, L., Vialet-Chabrand, S., Davey, P., Baker, N.R., Brendel, O., and Lawson, T. (2016). Effects of kinetics of light-induced stomatal responses on photosynthesis and water-use efficiency. New Phytol. *211*, 1209–1220.
18. Rui, Y., and Anderson, C.T. (2016). Functional analysis of cellulose and xyloglucan in the walls of stomatal guard cells of Arabidopsis. Plant Physiol. *170*, 1398–1419.
19. Mravec, J., Kračun, S.K., Rydahl, M.G., Westereng, B., Miart, F., Clausen, M.H., Fangel, J.U., Daugaard, M., Van Cutsem, P., De Fine Licht, H.H., et al. (2014). Tracking developmentally regulated post-synthetic processing of homogalacturonan and chitin using reciprocal oligosaccharide probes. Development *141*, 4841–4850.
20. Amsbury, S., Hunt, L., Elhaddad, N., Baillie, A., Lundgren, M., Verhertbruggen, Y., Scheller, H.V., Knox, J.P., Fleming, A.J., and Gray, J.E. (2016). Stomatal function requires pectin de-methyl-esterification of the guard cell wall. Curr. Biol. *26*, 2899–2906.
21. Verhertbruggen, Y., Marcus, S.E., Haeger, A., Ordaz-Ortiz, J.J., and Knox, J.P. (2009). An extended set of monoclonal antibodies to pectic homogalacturonan. Carbohydr. Res. *344*, 1858–1862.
22. Hervé, C., Rogowski, A., Gilbert, H.J., and Paul Knox, J. (2009). Enzymatic treatments reveal differential capacities for xylan recognition and degradation in primary and secondary plant cell walls. Plant J. *58*, 413–422.
23. Altartouri, B., and Geitmann, A. (2015). Understanding plant cell morphogenesis requires real-time monitoring of cell wall polymers. Curr. Opin. Plant Biol. *23*, 76–82.
24. Zhang, T., Zheng, Y., and Cosgrove, D.J. (2016). Spatial organization of cellulose microfibrils and matrix polysaccharides in primary plant cell walls as imaged by multichannel atomic force microscopy. Plant J. *85*, 179–192.
25. Jones, L., Milne, J.L., Ashford, D., and McQueen-Mason, S.J. (2003). Cell wall arabinan is essential for guard cell function. Proc. Natl. Acad. Sci. USA *100*, 11783–11788.
26. Franks, P.J., and Farquhar, G.D. (2007). The mechanical diversity of stomata and its significance in gas-exchange control. Plant Physiol. *143*, 78–87.
27. Franks, P.J., Cowan, I.R., and Farquhar, G.D. (1998). A study of stomatal mechanics using the cell pressure probe. Plant Cell Environ. *21*, 94–100.
28. Jones, L., Milne, J.L., Ashford, D., McCann, M.C., and McQueen-Mason, S.J. (2005). A conserved functional role of pectic polymers in stomatal guard cells from a range of plant species. Planta *221*, 255–264.

29. Lawson, T., and Blatt, M.R. (2014). Stomatal size, speed, and responsiveness impact on photosynthesis and water use efficiency. Plant Physiol. *164*, 1556–1570.

30. Franks, P.J., W Doheny-Adams, T., Britton-Harper, Z.J., and Gray, J.E. (2015). Increasing water-use efficiency directly through genetic manipulation of stomatal density. New Phytol. *207*, 188–195.

31. Dow, G.J., Bergmann, D.C., and Berry, J.A. (2014). An integrated model of stomatal development and leaf physiology. New Phytol. *201*, 1218–1226.

32. Li, B., Liu, G., Deng, Y., Xie, M., Feng, Z., Sun, M., Zhao, Y., Liang, L., Ding, N., and Jia, W. (2010). Excretion and folding of plasmalemma function to accommodate alterations in guard cell volume during stomatal closure in Vicia faba L. J. Exp. Bot. *61*, 3749–3758.

33. Meckel, T., Gall, L., Semrau, S., Homann, U., and Thiel, G. (2007). Guard cells elongate: relationship of volume and surface area during stomatal movement. Biophys. J. *92*, 1072–1080.

34. Veronda, D.R., and Westmann, R.A. (1970). Mechanical characterization of skin-finite deformations. J. Biomech. *3*, 111–124.

35. Maas, S.A., Ellis, B.J., Ateshian, G.A., and Weiss, J.A. (2012). FEBio: finite elements for biomechanics. J. Biomech. Eng. *134*, 011005.

36. Woolfenden, H.C., Bourdais, G., Kopischke, M., Miedes, E., Molina, A., Robatzek, S., and Morris, R.J. (2017). A computational approach for inferring the cell wall properties that govern guard cell dynamics. Plant J. http://dx.doi.org/10.1111/tpj.13640.

STAR★METHODS

KEY RESOURCES TABLE

REAGENT or RESOURCE	SOURCE	IDENTIFIER
Antibodies		
JIM7	http://www.plantprobes.net/index.php	JIM7
LM19	http://www.plantprobes.net/index.php	LM19
Cos488	[19]	Cos488
Anti-rat-IgG-FITC	Sigma-Aldrich	F6258-2ML
Chemicals, Peptides, and Recombinant Proteins		
Exo-polygalacturonase (6,500 U/mL)	Megazyme	E-PGALUSP
Endo-cellulase (700 U/mL)	Megazyme	E-CELTR
LR White Resin	London Resin Company	AGR1280
Experimental Models: Organisms/Strains		
Arabidopsis thaliana col-0	NASC; https://www.arabidopsis.org/portals/mutants/stockcenters.jsp	col-0
Software and Algorithms		
JPKSPM Data Processing software	JPK	JPK Instruments, DE; v. spm 5.0.69
FEBio	https://febio.org/	N/A

f-

CONTACT FOR REAGENT AND RESOURCE SHARING

Further information and requests for resources and reagents should be directed to and will be fulfilled by the Lead Contact, Andrew Fleming (a.fleming@sheffield.ac.uk).

EXPERIMENTAL MODEL AND SUBJECT DETAILS

Plant Growth and Tissue Treatments

For bioassay, immunolabeling and AFM experiments performed at Sheffield, Col-0 *Arabidopsis thaliana* seeds were stratified for 7 days at 4°C then germinated on 3:1 M3 compost:perlite in 6cm diameter, 8cm deep square pots and grown under 12hr light (200µmol m^{-2} s^{-1}) with 22°C day temperature, 16°C night temperature at 60% humidity. For AFM experiments performed at the Sainsbury Laboratory Cambridge University, Col-0 *Arabidopsis thaliana* seeds were grown on compost as above except growth conditions were 170µmol m^{-2} s^{-1}, 21°C day temperature, 17°C night temperature at 60% humidity. Leaves were taken from plants at 3-5 weeks for the various assays performed.

METHOD DETAILS

Stomatal Aperture Measurements

Epidermal peels of mature leaves were removed at least 2 hours into the photoperiod and floated onto opening buffer (50 mM KCl, 10 mM MES, pH 6.2). Samples were maintained at 22°C with 200 µmol m^{-2} s^{-1} of light. Air was bubbled into the opening buffer containing either 0 ppm CO_2 (CO_2 free treatment), ambient CO_2 or elevated CO_2 (1000 ppm) . Epidermal peels were imaged after 2 hours using an Olympus BX51 microscope and DP70 digital camera and stomatal apertures measured. For standard assays, 40 stomatal apertures were measured for each treatment in each of three independent experiments, with similar results beiong observed in each experiment. For each experiment epidermal peels were taken from at least 6 plants of each genotype. For enzyme treatments, dissected leaf blocks (approximately 5mm square) were treated for 4h at room temperature in buffer (10mM KCl, 0.1mM $CaCl_2$, 10mM MES pH 6.2,) containing exo-polygalacturonase (Megazyme, 6500U/ml) or endo-cellulase (Megazyme, 700U/ml) diluted 1/20 (v/v) in buffer, or incubated in buffer alone prior to analysis [28]. Calculation of theoretical maximal aperture was done according to [31] whereby $a_{max} = 0.25\pi.l^2$ where l = stomatal pore length under conditions of maximal opening (depleted CO_2).

Immunolabeling

For immunolabeling, leaf samples (3 mm diameter leaf discs) were fixed in 4% (w/v) formaldehyde in PEM buffer (0.1 M PIPES, 2 mM EGTA, 1 mM $MgSO_4$, adjusted to pH 7) by vacuum infiltration then dehydrated in an ethanol series (30 min each at 30%, 50%, 70%, 100% EtOH) and infiltrated with LR White Resin (London Resin Company) diluted in ethanol (45 min each at 10%, 20%, 30%, 50%, 70% & 90% resin then 3x8 h at 100%). Leaf discs were stacked vertically in gelatine capsules filled with resin and allowed to polymerize

or 7 days at 37°C. Sections were cut to a thickness of 2 μm using a Reichert-Jung Ultracut E ultramicrotome using a glass knife. Further processing and incubation with the JIM7 and LM19 antibodies was as previously described [20]. Briefly, sections were incubated with 3% (w/v) milk protein (Marvel, Premier Beverages, UK) in phosphate-buffered saline solution (PBS, pH 7.2) (hereafter known as PBS/MP). Sections were then incubated with a ten-fold dilution of primary monoclonal antibody in PBS/MP for 1 h at room temperature. Samples were washed 3 times with PBS and secondary antibody was added (100-fold dilution in PBS/MP) for 1 h. Samples were kept in the dark from this step. For the JIM- and LM- series of antibodies anti-rat-IgG (whole molecule) coupled to fluorescein isothiocyanate (FITC) was used. Samples were counterstained with 0.25% (w/v) Calcofluor White solution diluted ten-fold in PBS for 5 min before mounting on slides with Citifluor AF1 anti-fade solution (Agar Scientific, UK). Images were captured using a DP51 camera. FITC was visualized using a filter set with 460-490 nm excitation filter, a 510-550 nm emission filter and a 505 nm dichroic mirror. COS^{488} probe labeling of intact tissue samples was as previously described [19]. Briefly, fresh leaf tissue was submerged in a 1/1000 dilution of the COS^{488} probe in 50mM MES pH5.8 for 15minutes, then mounted in water for imaging on an Olympus FV1000 confocal microscope using a 488nm argon laser and an FITC filter set. Images were captured using Olympus Fluoview FV-ASW software.

Atomic Force Microscopy

Dissected and plasmolysed (0.55 M mannitol; minimum 45 min) leaf blocks (approximately 5mm square) from 3-4 week old plants were indented using a Nano Wizard 3 AFM (JPK Instruments, DE) mounted with a 5 nm diameter pyramidal indenter (Windsor Scientific, UK) on a cantilever of nominal 45 N/m stiffness. Cantilever stiffness was determined by thermal tuning prior to experiment initiation. Tip sensitivity was calibrated by first performing indentations on a clean glass slide, and varied between experiments. For each leaf, areas of 100x100 μm^2 were indented with 128x128 points on the adaxial surface. Indentations were performed with 1000 nN of force yielding an indentation depth range of 100-1000 nm. Sample numbers for each experiment are given in the figure legends and text. Force indentation curves were analyzed using JPKSPM Data Processing software (JPK Instruments, DE; v. spm 5.0.69) using the following steps: voltage readings were converted to force using calibrated sensitivity and cantilever stiffness values, baseline subtraction and tilt correction, vertical displacement offset adjustment, indentation calculation by subtraction of cantilever bending from piezo position during indentation, and indentation modulus was calculated by fitting a Hertzian indentation model to the approach curve. The Hertz model assumes the indented surface is an infinite homogeneous half space, which is clearly not the case for the geometrically complex leaf surface. Hence the results of indentation experiments are quoted as an apparent modulus, E_a. Control experiments carried out at lower indentation rates and at lower indentation depths revealed similar results, and analysis did not reveal any surface topography which might easily account for the E_a patterns observed around or within the guard cells. Retraction curves were not analyzed due to numerous adhesion difficulties during tip removal from the surface. All AFM images shown are derived from force maps, with an indication of the calculated E_a values according to the heatmaps adjacent to the images.

Modeling

Each guard cell is modeled as a hollow, deformed torus with ellipses describing the stomatal and pore outlines, and solid walls at the poles separating the two guard cells. In the baseline model, the cell wall thickness is uniform and is set to 1 μm (in the initial state) so that the guard cell cross-section is an annulus (Figure 2A). The model dimensions and cell wall thicknesses were set so that they matched observations for *Vicia faba* stomata [7, 32, 33]. The polar wall thickness in each guard cell is set to 0.3 μm. From the geometry, we used a custom script to create a mesh for the guard cells that was suitable for finite element calculations, which resulted in each guard cell being divided into approximately 20000 elements. We approximated the guard cell wall using the transversely-isotropic Veronda-Westmann material model [34] in FEBio [35] for our simulations. In short, this is an anisotropic elastic model that permits the independent paramerisation of the circumferential cellulose micro-fibrils (CMFs) and the isotropic cell wall matrix. The CMFs impart anisotropy to the cell wall. This material model is fully described in the documentation for FEBio with model cell wall parameters described in [36]. In the model, strain is a dimensionless tensor and characterizes the deformation of the material. Stress is a tensor which characterizes the internal forces within a material (force per area) in units of N/m^2 = Pa. FEBio uses the effective Lagrange strain and effective Cauchy stress to summarize these tensor measures.

QUANTIFICATION AND STATISTICAL ANALYSIS

For bioassay analyses, stomatal apertures were measured for each treatment in three independent experiments, with similar results beiong observed in each experiment. For each experiment epidermal peels were taken from at least 6 plants of each genotype. Data were analsysed by ANOVA with a post hoc Tukey analysis using commercial software (Graphpad 7) with significance being accepted at $p \leq 0.05$. The confidence limits and and sample size, n, for each experiment are given in the figure legends. For immunolabeling, experiments were performed at least three times on independent biological samples with similar results being observed.

For AFM, force curves were initially analyzed using JPKSPM Data Processing software (JPK Instruments, DE; v. spm 5.0.69). The number of force maps analyzed is stated in the appropriate figure legends. For the comparison of radial stiffness gradients a Mann-Whitney test was performed using the number of observations stated in the figure legend.

DATA AND SOFTWARE AVAILABILITY

AFM datasets, stomatal bioassay data and the guard cell FE model are available on request.

Current Biology, Volume *27*

Supplemental Information

Stomatal Opening Involves Polar, Not Radial,

Stiffening Of Guard Cells

Ross Carter, Hugh Woolfenden, Alice Baillie, Sam Amsbury, Sarah Carroll, Eleanor Healicon, Spyros Sovatzoglou, Sioban Braybrook, Julie E. Gray, Jamie Hobbs, Richard J. Morris, and Andrew J. Fleming

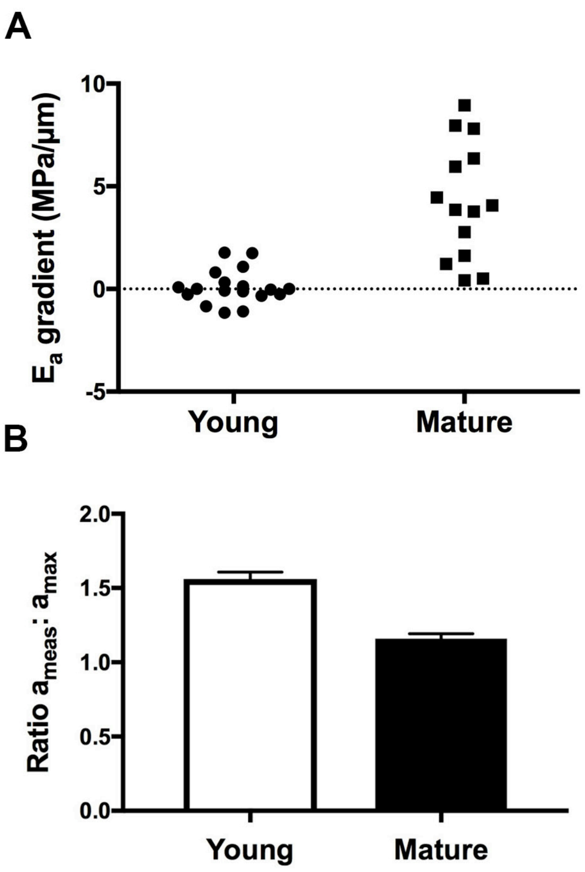

Figure S1. Related to Figure 1. Mature guard cells display a radial gradient in stiffness which is absent in young guard cells.

(A) Radial gradients (E_a/µm) calculated for individual guard cells. Stomata are classified as young (length:width ratio <1), giving guard cell n= 18, or mature (length:width ratio >1) giving guard cell n= 14. A Mann-Whitney test indicates that the mature guard cells displayed a significantly higher stiffness gradient than the young stomata ($p<0.001$). (B) Ratio of measured pore aperture (a_{meas}) to theoretical maximal aperture (a_{max}) for young or mature stomata after incubation under low CO_2 to open stomata. Error bars = sem.

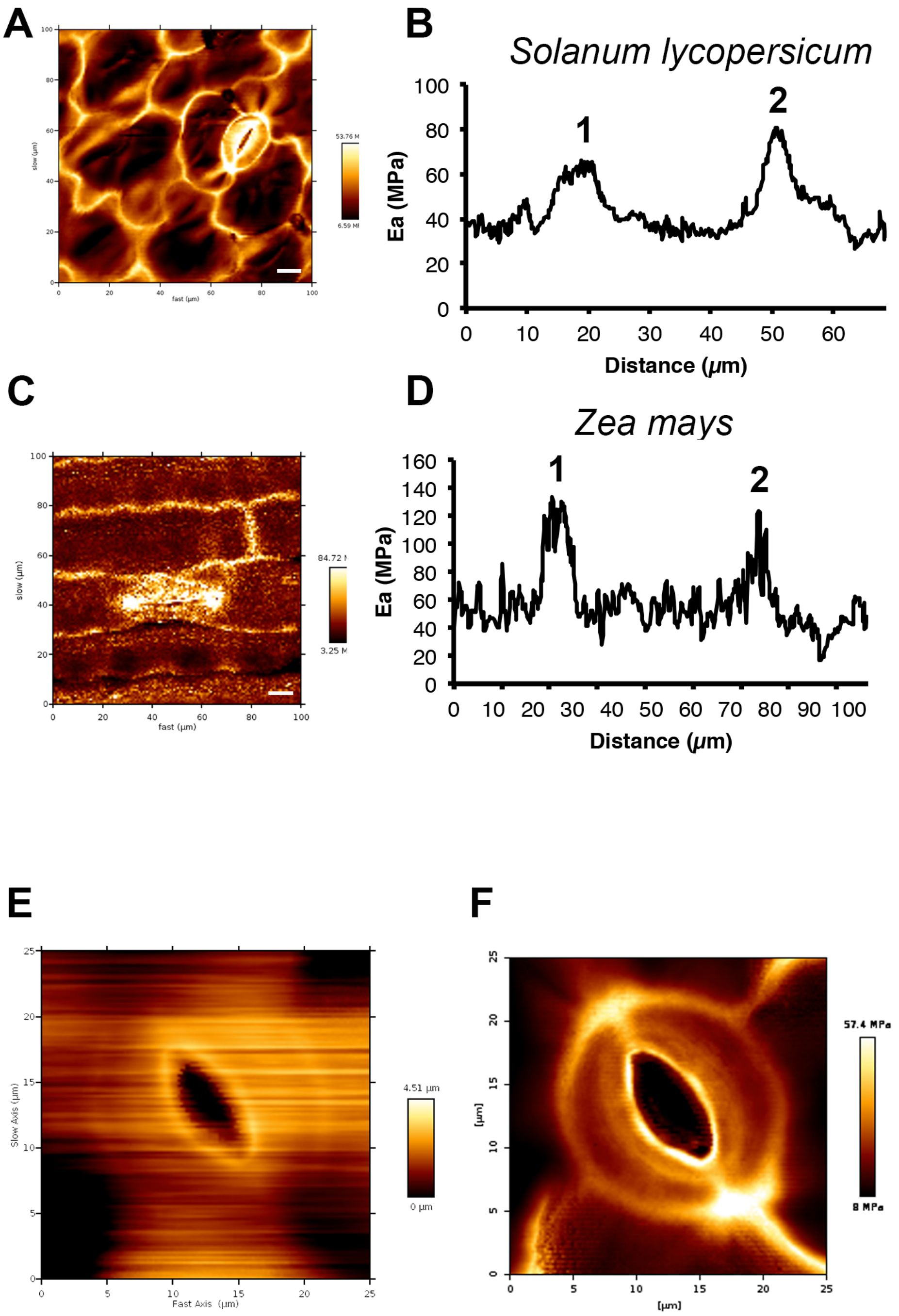

Figure S2. Related to Figure 1. Polar stiffening is observed in stomata from eudicot and monocot plants and does not reflect surface topography

(A) Force map of tomato (*Solanum lycopersicum*) epidermis showing the spatial pattern of Ea. (B) Distribution of Ea around the circumference of the stomatal complex shown in (A). Two main peaks of Ea are observed at the poles of the stomatal complex. (C) Force map of maize (*Zea mays*) epidermis showing the spatial pattern of Ea. (D) Distribution of Ea around the circumference of the stomatal complex shown in (C). Two main peaks of Ea are observed at the poles of the stomatal complex. Force maps and analyses presented are representative. Each analysis was performed at least three times with similar results. (E) Higher resolution topography and (F) Higher resolution force map of the Arabidopsis stomata shown in Figure 1I. In the vicinity of the guard cells there are no obvious topographical features corresponding to the polar localized regions of high Ea detected in (F). Scale bars A,C = 10µm.

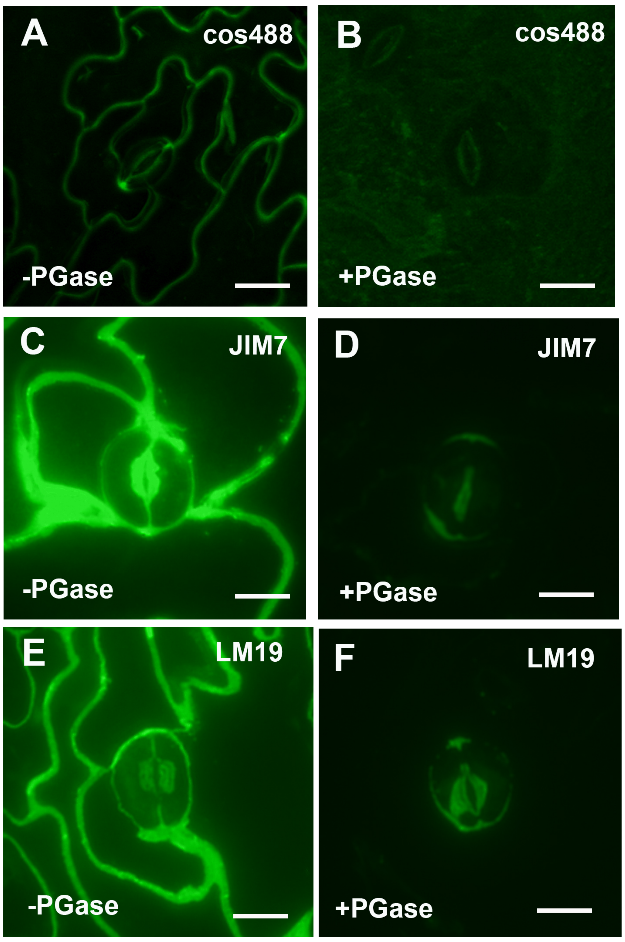

Figure S3. Related to Figure 4. Immunolabelling suggests that the stomatal poles have a specific pectin composition.

(A) Labeling with the COS^{488} probe reveals signal (green) in epidermal cell walls and peaks of signal intensity at the stomatal poles (as shown in Figure 4A) (B) Treatment of tissue with polygalacturonase (4h) leads to loss of COS^{488} binding throughout the epidermis. (C) Labelling with JIM7 antibody reveals signal (green) in all guard cell and epidermal walls, indicating the presence of pectin. (D) After treatment with polygalacturonase (4h) signal has disappeared from most walls but a weak signal is visible along the region of the cuticular ridge and in arcs around the stomatal poles. (E) Labelling with LM19 antibody, which detects de-esterified pectin, reveals a similar pattern to that observed in (C). (F) After treatment with polygalacturonase (4h) a pattern of LM19 binding similar to that observed in (D) is observed, with remnants of signal visible both along the inner radial walls and towards the stomatal poles. Images shown A-F are representative. Labellings were performed at least three times on independent biological samples with similar results being observed. Scale bars: A,B = 20 µm; (C-F) = 10 µm.

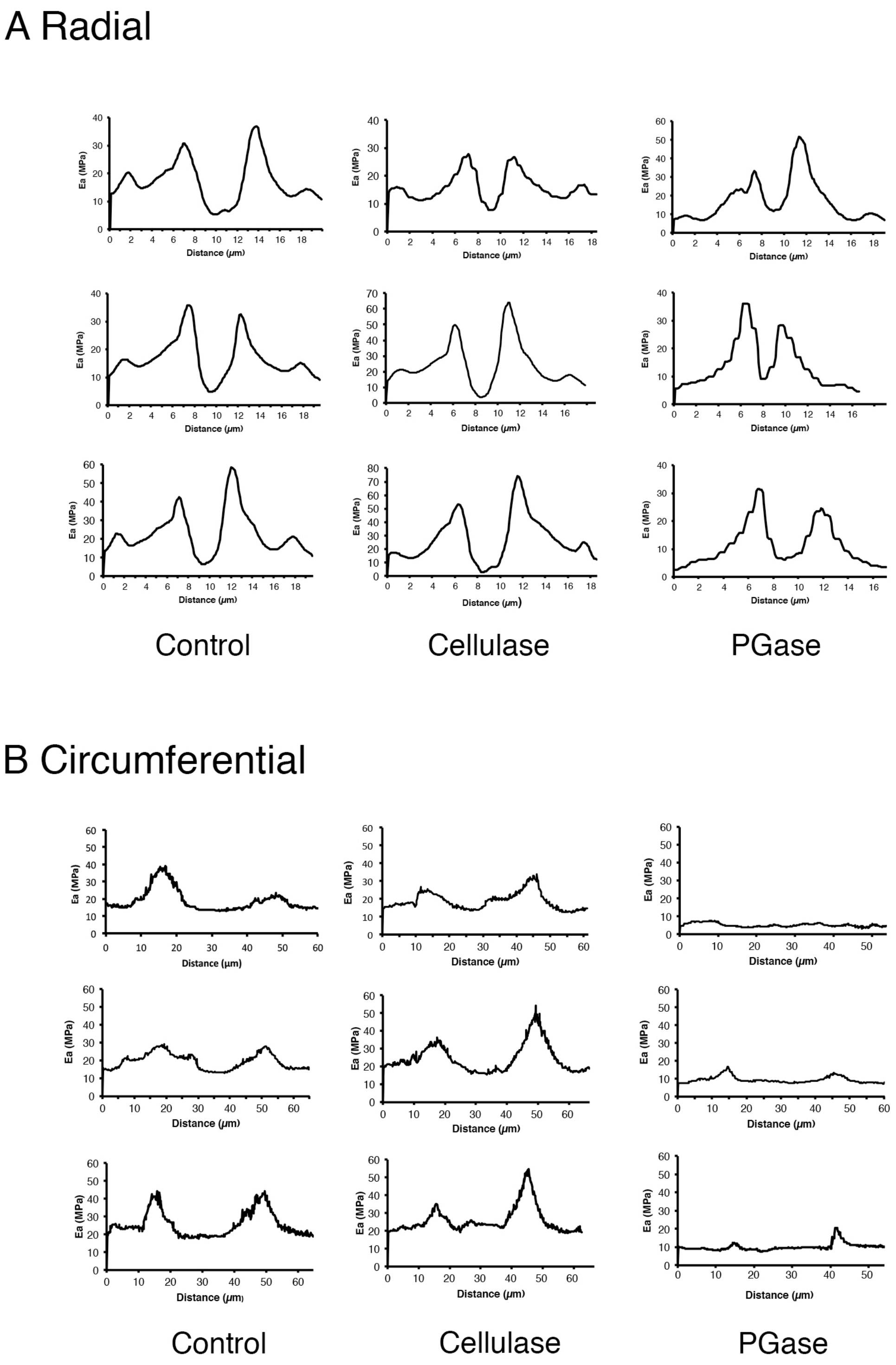

Figure S4. Related to Figure 4. Radial and circumferential Ea patterns in stomata treated with cell wall modifying enzymes

(A) Distribution of Ea across the diameter of the stomatal complexes (as indicated in schematic in Figure 1) after incubation for 4h in buffer (control), cellulase or polygalacturonase (PGase). Results are shown for 3 independent analyses for each treatment. The major peaks in Ea corresponding to the inner radial walls are clear in all samples but after PGase treatment the outer radial wall peaks are difficult to discern. (B) Distribution of Ea around the circumference (as indicated in schematic in Figure 1) of the stomatal complexes described in (A). Two main peaks of Ea corresponding to the poles of the stomatal complexes are apparent but after PGase treatment the peaks are more difficult to discern.
